# A Complex-Valued Self-Supervised Learning-Based Method for Specific Emitter Identification

**DOI:** 10.3390/e24070851

**Published:** 2022-06-21

**Authors:** Dongxing Zhao, Junan Yang, Hui Liu, Keju Huang

**Affiliations:** College of Electronic Engineering, National University of Defense Technology, Hefei 230031, China; zhaodongxing17@nudt.edu.cn (D.Z.); christ592604@163.com (H.L.); huangkeju@nudt.edu.cn (K.H.)

**Keywords:** specific emitter identification, signal processing, self-supervised learning, complex-valued neural network

## Abstract

Specific emitter identification (SEI) refers to distinguishing emitters using individual features extracted from wireless signals. The current SEI methods have proven to be accurate in tackling large labeled data sets at a high signal-to-noise ratio (SNR). However, their performance declines dramatically in the presence of small samples and a significant noise environment. To address this issue, we propose a complex self-supervised learning scheme to fully exploit the unlabeled samples, comprised of a pretext task adopting the contrastive learning concept and a downstream task. In the former task, we design an optimized data augmentation method based on communication signals to serve the contrastive conception. Then, we embed a complex-valued network in the learning to improve the robustness to noise. The proposed scheme demonstrates the generality of handling the small and sufficient samples cases across a wide range from 10 to 400 being labeled in each group. The experiment also shows a promising accuracy and robustness where the recognition results increase at 10–16% from 10–15 SNR.

## 1. Introduction

Specific emitter identification (SEI) distinguishes or identifies different emitters based on the “radio frequency (RF) fingerprint features” extracted from the received radio signals [1]. International research institutions have paid more and more attention to SEI because of its wide application in communication frequency band management, wireless network security, etc. [2,3]. However, the increasing complexity of the electromagnetic environment makes the task of SEI more and more difficult. For example, a low signal-to-noise ratio (SNR) environment may increase measurement loss or error, resulting in poor performance [4]. In order to suppress the influence of noise and improve the recognition accuracy, extensive research has been performed, such as using compressed sensing reconstruction algorithms to recover structured signals [5,6,7,8], as well as using complex-valued neural networks to improve the anti-noise performance [9]. Although the existing methods improve the signal quality, with the increasing cost of labeled samples, the methods cannot complete SEI well with small samples. Therefore, SEI has become an urgent and challenging issue.

The traditional SEI method is mainly based on manual feature extraction, which includes three steps. Step 1: select appropriate “RF fingerprint features” using certain training samples and expert knowledge. Step 2: extract the “RF fingerprint features” in the signal processing stage. Step 3: distinguish individual emitters through the extracted features. The traditional SEI method mainly extracts the features of two kinds of signals for classification: transient and steady-state signals.

The research direction of SEI methods based on transient signals is mainly in signal detection and feature extraction. At this stage, the research on signal detection specifically includes the Markov model [10], naive Bayes [11], and wavelet transform [12]. Signal detection mainly distinguishes signals from signal energy. We can also identify the signal from the features of the signal itself, such as frequency and bandwidth. Typical artificial feature extraction methods include generalized size representation [13] and wavelet transform [14]. However, the performance of transient signal recognition depends on signal detection accuracy [15]. In other words, the excessive error in transient signal detection will lead to the poor performance of the SEI method based on transient signals. Therefore, a large number of features based on steady-state signals have been proposed, including bispectrum [16], cumulant [17], wavelet transform [18], short-time Fourier transform [19], Hilbert–Huang transform [20], and Wigner–Ville transform [21]. However, these traditional SEI methods rely heavily on expert knowledge and prior knowledge [22].

In the past few years, deep learning has shown its effect in computer vision [23,24] and natural language processing [25]. Recently, various deep learning network frameworks have proven to have great potential in computer vision, such as convolution neural networks [26], residual networks (ResNets) [27], generation countermeasure networks [28], and recurrent neural networks [29]. Moreover, deep learning methods have been fully developed in the field of emitter recognition, which can be divided into two categories. One is the convolutional neural-network-based method for SEI [30], which mainly focuses on the amplitude and phase information of the signal. For example, in [31], the author takes the envelope in front of the radar signal as the network’s input. The envelope characteristics of the individual emitter are automatically extracted through the network, so as to fit the Q-value of the current state action. The literature also discusses the effects of the deep Q network model, deep double Q network model, and dueling network model in emitter recognition. The other is the recurrent neural-network-based method for SEI [29], which mainly focuses on the temporal information of the signal. For example, in [32], the author uses a bidirectional short-term memory network to extract time structure features from the baseband’s in-phase signal and quadrature-phase signal (I/Q). The method based on deep learning effectively makes up for the limitation that the traditional SEI method needs expert knowledge. However, both methods based on deep learning and manual feature extraction need a large number of labeled samples. As the electromagnetic environment becomes more and more complicated, manual tagging becomes more costly, which leads to the fact that most samples are still unlabeled. In other words, the existing methods only uses a small number of labeled samples to train the network, which cannot meet the requirements of recognition accuracy and does not make full use of unlabeled samples. Therefore, we propose an SEI method based on complex-valued self-supervised learning, which tries to solve the problem of low recognition accuracy with small samples.

In recent years, self-supervised learning has made great breakthroughs in the field of natural language processing [33], such as bidirectional encoder representation from transformers (BERT) [34] and generative pre-training (GPT) [35]. Self-supervised learning is generally divided into two stages: pretext and downstream tasks. In the past, because the original signal was in a continuous high-dimensional space, unsupervised learning could not work effectively in computer vision, resulting in the supervised networks still being dominant. Recently, some self-supervised models (such as SimCLR [36], SwAV [37], and Moco [38]) have been proposed successfully, which proves that self-supervised learning can achieve the same effect as supervised learning in the field of computer vision [39].Using the idea of contrastive learning for reference, we apply self-supervised learning to SEI to solve the problem of low recognition accuracy with small samples. In view of the fact that the number of labeled samples in the data set is far less than that of unlabeled samples, we use the contrastive learning of different enhanced views of a large number of unlabeled samples to train a new and potentially augmented representation in the pretext task. Then, in the downstream task, SEI can be performed by fine-tuning the encoder network with a small number of label samples.

In this paper, a complex-valued self-supervised learning-based for SEI is proposed. We effectively combine complex-valued networks and self-supervised learning in SEI. Firstly, we use many unlabeled samples to pre-train the network in the stage of the pretext task, which is used to obtain the initial weight of the complex-valued encoder. Secondly, in the stage of the downstream task, we use a small number of labeled samples to fine-tune the weight of the complex-valued encoder. Finally, the trained complex-valued encoder is used to perform SEI. The main contributions of this article are summarized as follows:

- We apply the self-supervised learning framework to SEI, which solves the problem of low recognition accuracy with small samples. Self-supervised learning includes two tasks: pretext task and downstream task. In the pretext task, we can directly use unlabeled samples for contrastive learning, which increases the utilization of unlabeled samples. Our proposed method can achieve 84% recognition accuracy in the downstream task, even if the number of labeled samples of each type of emitter is 25.
- We first combine the complex-valued neural network with self-supervised learning. Using the stage of pretext task to pre-train the complex-valued encoder to obtain the initial value of the weight solves the problem that the complex-valued network is sensitive to the weight initialization. In addition, the high anti-noise performance of complex-valued networks is used to improve the robustness of the self-supervised learning method to noise.
- The data augmentation methods used in self-supervised learning are mainly related to images, but are not suitable for signals. Therefore, we propose three new data augmentation methods based on communication signals: phase rotation, random cropping, and jitter.

The rest of this paper is organized as follows: The second section briefly introduces the system model of SEI and its four key steps. The third section introduces self-supervised learning and the complex-valued neural network. The fourth section provides the design details of the proposed method. The fifth section discusses the comparative experimental results on real data sets. The sixth part summarizes this paper.

## 2. System Model

In this section, we briefly introduce the specific emitter identification system model in the order of signal acquisition, data pre-processing, feature extraction, and classification.

### 2.1. Signal Acquisition

**Figure 1:**
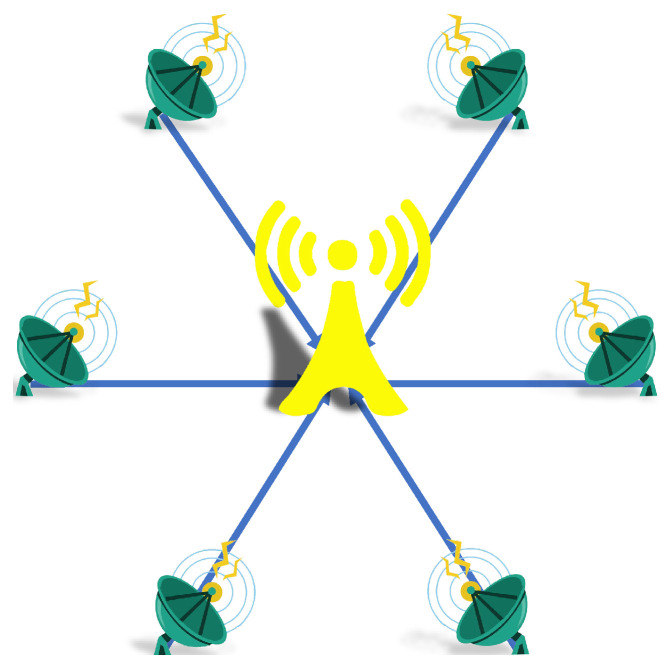
The model of signal acquisition.

As shown in Figure 1, there are *N* identical emitters and a quadrature receiver in an open space. It is assumed that only one of the *N* emitters is active at a time, which means the signal of each emitter can be captured separately. The transmitted signal can be expressed as:

(1) r(t)=Acos(2πft+φ),

where *A* is the signal’s amplitude and φ is the signal’s phase. In signal transmission, both the channel and noise will affect it. The received signal can be expressed as:

(2) $$s\left(t\right)=h\left(t\right)*f_{k}\left(r\left(t\right)\right)+n\left(t\right),k\in \left[1,2,3\cdots ,N\right]$$

where h(t) is the channel response. n(t) is noise. “∗” represents convolution. $f_{k}$ is the individual characteristics of the emitters. The receiver samples the signal at the sampling frequency of FS and outputs two signals, namely in-phase and quadrature-phase signals (I/Q). The sampled signal can be expressed as:

(3) $$s\left(n\right)=\sqrt{s_{i}^{2}\left(n\right)+s_{q}^{2}\left(n\right)},$$

where $s_{i}^{2}\left(n\right)$ is the in-phase signal and $s_{q}^{2}\left(n\right)$ is the quadrature-phase signal. We divided the sampled data sets into two categories according to whether they are labeled or not. One is unlabeled data sets $X=\{x_{1},x_{2},\cdots ,x_{m}\}$, which have a large number of samples and have not been labeled manually. The other one is labeled data sets $S=\{s_{1},s_{2},\cdots ,s_{n}\}$, which have a small number of samples and have been manually labeled. The labels are $L=\{l_{1},l_{2},\cdots ,l_{n}\}$. We used many unlabeled samples to train the encoder in the stage of the pretext task to obtain the initial weight value. Then, in the stage of the downstream task, a small number of labeled samples were used to fine-tune the weight of the encoder network to complete the migration learning.

### 2.2. Data Pre-Processing

The distribution of actual sampling data is often uneven due to noise and other factors. In other words, the eigenvalues of data sets are often a non-Gaussian distribution. Therefore, we need to convert data from different distributions to the same distribution, that is standardize the data. The formula is as follows:

(4) $$x^{\prime }=\frac{x-\mu }{\sigma },$$

where μ is the mean of data *x* and σ is the standard deviation. Through data standardization, we can not only speed up the solution speed of the network model, but also improve the accuracy of the model and, finally, accelerate the convergence of the model.

### 2.3. Feature Extraction and Classification

**Figure 2:**
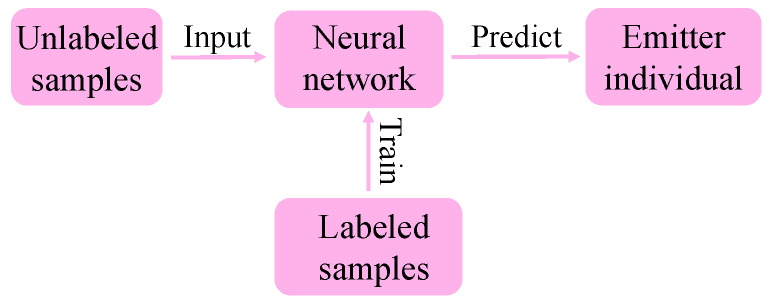
Feature extraction method based on self-supervised learning.

The self-supervised learning method is different from the traditional SEI method, which requires manual feature extraction. It can automatically learn the critical information in the data and extract the basic features. Figure 2 shows the feature extraction method based on self-supervised learning.

Firstly, we used a large number of unlabeled samples to pre-train the network. Secondly, a small number of labeled samples were used to fine-tune the network weight. Finally, the trained network was introduced to classify and identify individual emitters.

## 3. Related Work

Most of the SEI methods need many manual labeled samples, which requires much human and material cost, so it is challenging to meet the practical requirements. Therefore, unsupervised learning methods such as self-supervised learning have received paid more and more attention. Self-supervised learning usually includes two stages: pretext task and downstream task. In the stage of the pretext task, the network pre-trains the encoder by constructing auxiliary tasks. In the stage of the downstream task, the weight parameters of the encoder are fine-tuned to perform SEI. In this section, we review some widely used methods related to the work of this paper.

### 3.1. Pretext Task

The mainstream pretext tasks are roughly divided into three categories: context-based, temporal-based, and contrastive-based. Encoders can learn the essential data representation in many unlabeled samples to be qualified for downstream tasks through various data augmentation methods. The contrastive-based method mainly trains the encoder network by increasing the similarity between positive samples and reducing the similarity between positive and negative samples, which is the most popular method in self-supervised learning. In other words, the contrastive-based method makes the distance between positive samples far less than that between positive and negative samples [40]. The formula is as follows:

(5) $$F\left(f\left(x\right),f\left(x^{+}\right)\right)\gg F\left(f\left(x\right),f\left(x^{-}\right)\right),$$

where f(•) represents the output of the encoder. F(•) is the similarity measure function. $x^{+}$ and $x^{-}$ are positive and negative samples of *x*, respectively.

### 3.2. Loss Function

The loss function is usually used as the link between learning criteria and optimization problems. That is, the model is evaluated by minimizing the loss function. This is a critical evaluation index of the training encoder. Generally, three methods define the loss function: mean-squared error, cross-entropy, and self-defined loss function.

As a variant of cross-entropy, contrastive loss’s target can be defined according to the data representation calculated by the network, rather than matching the input with the fixed target [41]. It is the most popular loss function in the field of unsupervised learning recently [42,43], and the specific details will be described in the next section. In unsupervised learning, there is also a loss function called adversarial loss, which is mainly used for the generation of unsupervised data [44].

### 3.3. Complex-Valued Neural Network

At present, complex-valued neural networks have achieved comparable or even better results than real-valued networks in some fields, such as music processing [9] and wireless communication [45]. The difficulties of the complex-valued neural network are complex convolution and complex activation function. Compared with real numbers, the differentiability of convolution and the activation function was not achieved until the emergence of [9]. The formula of complex convolution is as follows:

(6) W∗v=(A∗x−B∗y)+i(B∗x+A∗y),

where *A* and *B* are real matrices. *x* and *y* are real vectors. The formula takes the difference of two parallel inputs as a new real part and the sum of inputs as a new imaginary part so that the network can extract the phase information of complex-valued signals.

Reference [9] proposed three complex-valued activation functions, namely CReLU, modReLU, and zReLU. Their expressions are as follows:

(7) $$modReLU\left(z\right)=ReLU\left(\left|z\right|+b\right)e^{i\theta_{z}}=\left\{\begin{array}{cc} \left(\left|z\right|+b\right)\frac{z}{\left|z\right|} & \left|z\right|+b\geq \;0\\ 0 & otherwise \end{array}\right.$$

(8) CReLU(z)=ReLU(ℜ(z))+iReLU(ℑ(z))

(9) $$zReLU\left(z\right)=\left\{\begin{array}{cc} z & \theta_{z}\in \left[0,\pi /2\right]\\ 0 & otherwise \end{array}\right.$$

Compared with the other two activation functions, CReLU performs better in music processing [9]. Therefore, our later experiments also use this activation function. The complex-valued network can fully mine the correlation between I/Q signals and has high robustness to complex plane noise. In other words, the complex-valued network can have better recognition accuracy under a low signal-to-noise ratio. However, experiments have shown that complex-valued neural networks are susceptible to the initialization of weights [46]. To solve this problem, we combined self-supervised learning with the complex-valued neural network and initialize the weight of the complex-valued network through the pretext task stage of self-supervised learning. Firstly, we used the pre-training in the stage of the pretext task to obtain the initial weight value of the network to avoid the negative impact of random weight initialization. Secondly, we used the excellent anti-noise performance of the complex-valued network to improve the robustness of the network to noise.

## 4. Methodology

A main purpose of self-supervised learning is to pre-train representations (i.e., features) that can be transferred to downstream tasks by fine-tuning, whose core is contrastive learning, which can be considered as building dynamic dictionaries. The “keys” (tokens) in the dictionary are sampled from data (e.g., images or signal samples) and are represented by an encoder network. Unsupervised learning trains encoders to perform dictionary look-up: an encoded “query” should be similar to its matching key and dissimilar from others. Learning is formulated as minimizing a contrastive loss (discussed later in context).

**Figure 3:**
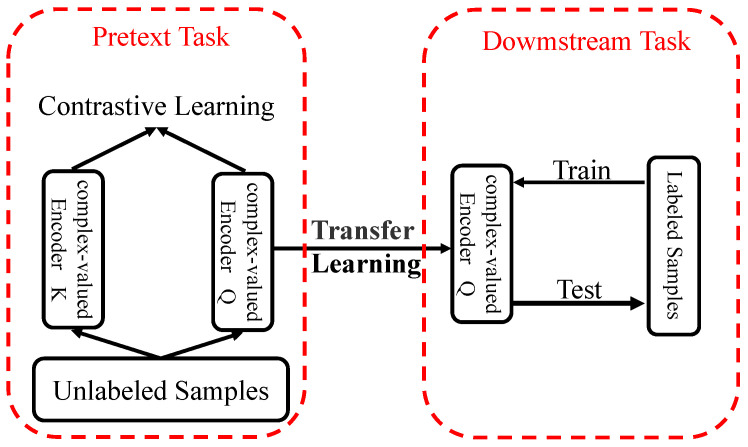
The model of complex-valued self-supervised learning.

We combined the complex-valued neural network with self-supervised learning to improve the model’s performance. In the stage of the pretext task, the complex-valued encoder is trained by contrastive learning to obtain the initial weight value of the network. In the stage of the downstream task, a small number of labeled samples are used to fine-tune the weight of the complex-valued encoder to perform SEI. Figure 3 shows the model of complex-valued self-supervised learning. This section will focus on the two critical stages of self-supervised learning: pretext task and downstream task. We also introduce three new data augmentation methods and the loss function.

### 4.1. Pretext Task

**Figure 4:**
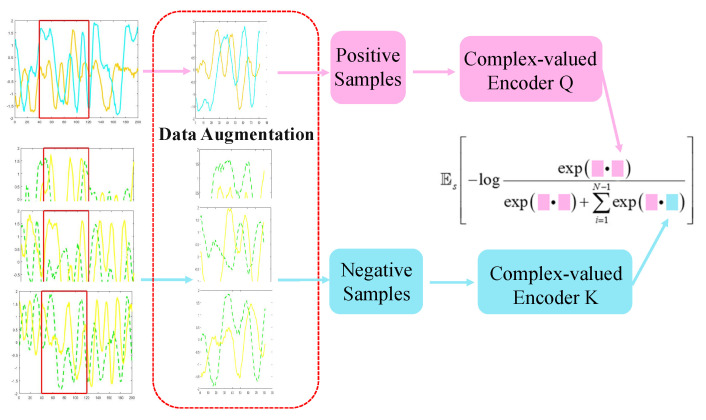
Pretext task.

Figure 4 shows the pretext task of the complex-valued self-supervised model. Firstly, we generated positive and negative samples from a batch of samples through three new data augmentation methods. Secondly, we input the positive samples into the complex-valued encoder Q and the negative samples into the complex-valued encoder K to generate the feature vector of the samples. Finally, the iterative update of the network is carried out through the contrastive learning of the feature vectors of positive and negative samples. The complex-valued encoder Q is updated by the gradient descent algorithm. The complex-valued encoder K updates the model parameters through the momentum update method.

### 4.2. Data Augmentation

We propose three new data augmentation methods to generate positive and negative samples: phase rotation, random cropping, and jitter.

Phase rotation aims to multiply the complex signal by a random phase to achieve the purpose of data expansion. Its formula is as follows:

(10) $$s{\left(t\right)}^{\prime }=s\left(t\right)e^{j\theta },$$

where the original signal s(t) is a complex signal. $s{\left(t\right)}^{\prime }$ is the signal after phase rotation. θ is a constant that obeys the uniform distribution of $\left[0,2\pi \right]$. Through phase reversal, we can change the phase of the I/Q signals to simulate the random phase jitter in signal transmission.

Random cropping is a standard method of data augmentation. Random cropping usually removes or masks a part of the picture in the image field and constructs a task to make the network restore the cropped part of the picture. The difference between the signal and image is that the characteristics of the signal are continuous. In other words, to maintain the integrity of “RF fingerprint features”, we must keep the continuity of the signal. However, the randomly cropped images are often discontinuous. Therefore, different from the random cropping in the image, the signal samples after cropping are continuous. To further meet signal processing requirements, we cut the signal into a fixed length.

Jitter expands the data by adding additive Gaussian white noise to make the model more robust. The expression of jitter is as follows:

(11) $$s{\left(t\right)}^{\prime }=s\left(t\right)+N\left(t\right),$$

where N(t) is additive Gaussian white noise added to the test samples. Its mean value is 0, and its variance satisfies the uniform distribution of $\left(0,1\right)$.

### 4.3. Build a Queue

In general, contrastive learning in the pretext task mainly depends on the number of positive and negative samples. If we input negative samples into the network by batch, we must set a larger batch size to improve the effect of contrastive learning, which may be harmful to the network. Because the size of the batch cannot be too large or too small [47], we input negative samples into the network in the form of a queue rather than a batch, which can separate the number of negative samples from batch size. In addition, results show that although the queue length is set much larger than the batch size, we can still easily set the super parameters. Therefore, we propose to store the negative samples generated by different batches in the form of queues. The current batch is enqueued, and the oldest batch in the queue is removed. Since the encoding key of old data is outdated, it is beneficial for the network to delete it.

### 4.4. Momentum Update

**Figure 5:**
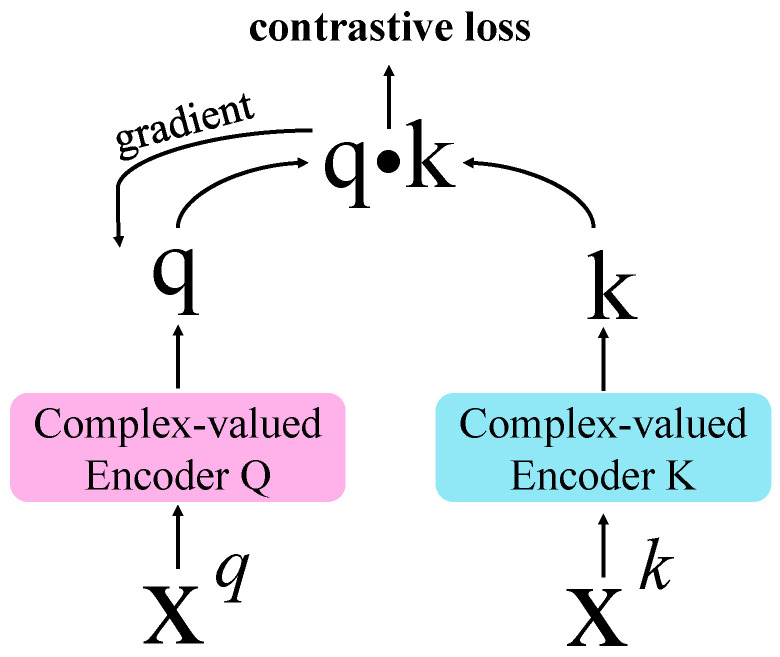
Momentum update.

Reference [38] found that storing negative samples in the queue can improve the effect of contrastive learning, but it will make the backpropagation of gradients more difficult. Therefore, a momentum-updating method is proposed to solve this problem. As shown in Figure 5, for the complex-valued encoder Q, we still used the general gradient descent algorithm to update the weight of the encoder. For the weight of complex-valued encoder K, we updated them with the following formula:

(12) $$\theta_{k}\leftarrow m\theta_{k}+\left(1-m\right)\theta_{q},$$

where $\theta_{k}$ represents the parameters of encoder K. $\theta_{q}$ represents the parameter of encoder Q. *m* is the momentum coefficient, whose value is [0,1). Experiments show that when the number of *m* is large, the result is better, which shows that slowly updating encoder K is conducive to making full use of all negative samples in the queue.

### 4.5. Loss Function

As the objective function of unsupervised learning, the contrastive loss is the key to the iterative update of the query encoder and key encoder networks. In general, $q=f_{q}\left(X^{q}\right)$ represents the query, where $f_{q}$ is the encoder network and $X^{q}$ is the query sample. Similarly, $k=f_{k}\left(X^{k}\right)$ represents the key of the dictionary. Suppose there is now an encoded query *q* and a set of dictionary keys $\left\{k_{0},k_{1},k_{2},\cdots \right\}$, in which only one key $k_{n}$ matches *q*. That is, the contrastive loss function should have a lower value when *q* is similar to $k_{n}$ and not similar to other keys. This paper considers a loss function called InfoNCE [43], which uses the point product to measure similarity. The formula is as follows:

(13) $$L_{N}=E_{s}\left[-\log\frac{exp(q•k_{n}/\tau )}{\sum_{j=0}^{N}exp\left(q•k_{j}/\tau \right)}\right]$$

where τ is the temperature super parameter [42]. “=‘•” is the dot product of vectors. InfoNCE calculates the sum of the dot product of a positive sample and *N* negative samples. By reducing the value of InfoNCE, we can increase the similarity between *q* and $k_{n}$ and lessen the similarity between *q* and other keys.

### 4.6. Downstream Task

Firstly, we transferred the complex-valued encoder Q, which has completed the pre-training. Secondly, we used a small number of labeled samples to fine-tune the weight of complex-valued encoder Q. Finally, we used encoder Q to classify and identify samples of unknown emitters. Since the complex-valued encoder Q has learned the essential feature representation from many unlabeled samples, we only need a small number of labeled samples to obtain high recognition accuracy, which solves the problem of low recognition accuracy with small samples.

## 5. Experiments

In this section, we introduce the parameter setting of our data set and network model and discuss the superior performance of our proposed method compared with other SEI methods.

### 5.1. Data Set and Parameter Setting

The electromagnetic signal was collected from 8 emitters and a quadrature receiver. The carrier frequency of the emitter was 50 MHz. The sampling frequency of the receiver was 50 MHz. The number of sampling points was 16,384, of which the first 8192 points were in-phase signals and the last 8192 points were quadrature-phase signals.

We used PyTorch as the framework and Adam as the optimizer. In the stage of the pretext task, the learning rate was $10^{-5}$. The batch size was 32. The epoch was 500. The temperature hyperparameter was 0.05. The momentum value was 0.05. The length of the queue was 50. We used unlabeled samples for model pre-training, in which there were 10,000 unlabeled samples for each type of emitter. In the stage of the downstream task, the learning rate was $10^{-2}$. The batch size was 64. The epoch was 1000. We used labeled samples to fine-tune the weight of the migrated encoder Q, including 400 training samples and 400 test samples for each type of emitter.

### 5.2. Results

We tested our model from three aspects. Firstly, we compared our model with the traditional complex-valued and real-valued networks. Secondly, we tested the recognition accuracy of the model under different numbers of samples after artificially adding noise to the test samples. Finally, we tested the recognition accuracy of the model under different signal-to-noise ratios.

We tested the recognition accuracy of each model under the condition of small samples (the number of samples was 10, 15, 20, and 25) and sufficient samples (the number of samples was 200 and 400), as shown in the following Table 1.

CSSL represents complex-valued self-supervised learning. CVNN represents the complex-valued neural network. RSSL represents real-valued self-supervised learning. RDAN represents real-valued data augmentation network. RRN represents the real-valued residual network. We took the average of five experiments as the final value of our recognition accuracy. To prove that our method solves the acute problem of weight initialization in complex-valued neural networks [46], we also put the standard deviation of five experimental results in brackets in the table. It can be seen from the table that the network performance of the complex-valued neural network is not particularly sensitive to weight initialization when the number of samples is sufficient. However, when the number of samples is small, once the weight initialization is inappropriate, the network accuracy will deteriorate sharply and even cannot meet the requirements of SEI. Therefore, we used a large number of unlabeled samples to fully pre-train the network so that the network has a stable and appropriate initial value. In addition, sufficient pre-training can also make the network have better recognition accuracy. When compared with real-valued self-supervised learning, we can see that the complex-valued self-supervised learning network can improve the recognition accuracy of the network, whether in the condition of small samples or sufficient samples.

To simulate the influence of noise on signal transmission, we added additive Gaussian white noise to the test samples, reduced its signal-to-noise ratio to 10 dB, and carried out experiments under different sample numbers. The results are shown in Table 2 below.

It can be seen from the table that our proposed method has better anti-noise robustness. Especially under small samples, complex-valued self-supervised learning can improve the performance of real-valued self-supervised learning by more than 10%. Compared with the data in Table 1, we can see that when the signal-to-noise ratio (SNR) is reduced by 5 dB and the samples are sufficient, the accuracy of our method is reduced by about 5%. When the number of samples is small, the accuracy decreases by about 10%, which means that the complex-valued self-supervised network is more sensitive to noise with small samples. In other words, when the number of samples is small, the change in SNR has a more significant impact on the network.

To further simulate the influence of noise on each model, when the number of training samples is 400, we tested the recognition accuracy of each model under different signal-to-noise ratios. The results are shown in Table 3.

It can be seen from the table that with the decrease in the signal-to-noise ratio, the recognition accuracy of each model will decline. However, the complex-valued self-supervised learning method has higher recognition accuracy under a low signal-to-noise ratio compared with other traditional methods. In particular, compared with the real-valued self-supervised learning method, the accuracy of the complex-valued self-supervised learning method can be improved by nearly 5%, which proves the effectiveness of our proposed method. Results show that the anti-noise performance of self-supervised learning can be significantly enhanced by combining complex-valued neural networks with self-supervised learning.

## 6. Conclusions

This paper combined the complex-valued neural network with self-supervised learning and proposed a complex-valued self-supervised learning-based method for SEI. The method consists of two stages: pretext task and downstream task. In the stage of the pretext task, we used a large number of unlabeled samples to pre-train the complex-valued encoder through contrastive learning. In the stage of the downstream task, we used a small number of labeled samples to fine-tune the weight of the encoder to perform SEI. The experimental results showed that the complex-valued self-supervised learning method can not only solve the problem that the complex-valued neural network is sensitive to weight initialization with small samples, but also improve the robustness of self-supervised learning to noise. Compared with some existing traditional methods, our proposed method can have higher recognition accuracy both under the condition of small samples or sufficient samples. However, the complex-valued neural network needs more parameters than the real-valued neural network, leading to a longer training time than the real-valued network. In the future, we will improve the method through a lightweight network. The implicit assumption in this paper is that the training and test data satisfy the independent identically distributed condition. When this assumption is not met, the recognition accuracy of the network will be significantly reduced. The possible way to solve this problem is to develop adaptive methods with loose assumptions, such as partial domain adaptation. Applying these methods to SEI is our future work.

## Figures and Tables

**Table 1:** Recognition accuracy under signal-to-noise ratio of 15 dB.

	Number	10	15	20	25	200	400
Acc (%)							
CSSL		**70.09 (±3.8)**	**75.13(±2.0)**	**77.56 (±2.6)**	**84.56 (±2.1)**	**93.87 (±1.5)**	**98.34 (±1.0)**
CVNN		55.80 (±13.8)	63.20 (±10.8)	58.74 (±9.3)	66.68 (±5.4)	86.34 (±3.9)	92.12 (±2.4)
RSSL		68.13	72.00	75.21	79.62	90.91	93.06
RDAN		55.09	60.62	67.09	76.84	89.84	91.00
RRN		23.65	25.18	26.28	27.50	48.18	65.65

**Table 2:** Recognition accuracy under signal-to-noise ratio of 10 dB.

	Number	10	15	20	25	200	400
Acc (%)							
CSSL		**61.78**	**66.03**	**68.75**	**70.65**	**84.84**	**93.88**
CVNN		50.56	51.57	52.24	56.50	79.25	83.18
RDAN		50.34	50.47	51.25	51.53	76.34	81.37
RRN		19.18	19.37	23.43	25.93	37.56	40.90

**Table 3:** Recognition accuracy under different SNRs.

	SNR	3	4	5	6	7
Acc (%)						
CSSL		**85.28**	**86.28**	**89.25**	**92.06**	**90.31**
CVNN		83.15	83.49	84.72	85.18	85.78
RDAN		81.37	82.43	83.71	83.84	86.34
RRN		16.43	24.62	30.65	32.22	34.65

## Data Availability

Not applicable.
